# Editorial: Reproducibility in neuroscience

**DOI:** 10.3389/fnint.2023.1271818

**Published:** 2023-08-25

**Authors:** Nafisa M. Jadavji, Nele A. Haelterman, Reeteka Sud, Alberto Antonietti

**Affiliations:** ^1^Department of Biomedical Sciences, College of Graduate Studies, College of Veterinary Medicine, College of Osteopathic Medicine, Midwestern University, Glendale, AZ, United States; ^2^Department of Child Health, College of Medicine Phoenix, University of Arizona, Phoenix, AZ, United States; ^3^Department of Neuroscience, Carleton University, Ottawa, ON, Canada; ^4^Department of Molecular and Human Genetics, Baylor College of Medicine, Houston, TX, United States; ^5^Center for Brain and Mind, Department of Psychiatry, NIMHANS, Bengaluru, Karnataka, India; ^6^Department of Electronics, Information and Bioengineering, Politecnico di Milano, Milan, Italy

**Keywords:** replicability, FAIR (findable accessible interoperable and reusable) principles, rigor and quality, research integrity, replication studies

Scientific progress depends on the ability to independently repeat and validate key scientific findings. To verify the rigor, robustness, and validity of a research study, scientists test if they can reach the same conclusions when they use the same methods, data, and code (reproducible result) or when they use a different, independent model, technology, or tool (replicable result). Several large-scale efforts have revealed significant challenges related to reproducing and replicating research studies, including lack of access to research reagents, detailed methodology, or source code developed for the study (Manninen et al., 2018; Errington et al., 2021; Botvinik-Nezer and Wager, 2022). In addition, studies that “merely” repeat a published work are seen as lacking novelty and, therefore, difficult to fund and publish, further lowering the incentive for researchers to embark on replication or reproducibility studies, but this is starting to change.

Several organizations worldwide have tried to increase awareness about the importance of reproducibility and replicability in different disciplines in recent years. Myriad tools have been developed to support rigor and reproducibility, including open-source repositories for research resources, protocols, source code, data, etc. (Figure 1). We initiated this Research Topic to highlight how these tools promote efforts to replicate basic and computational research studies in integrative neuroscience and to increase awareness of this vital topic.

**Figure 1:**
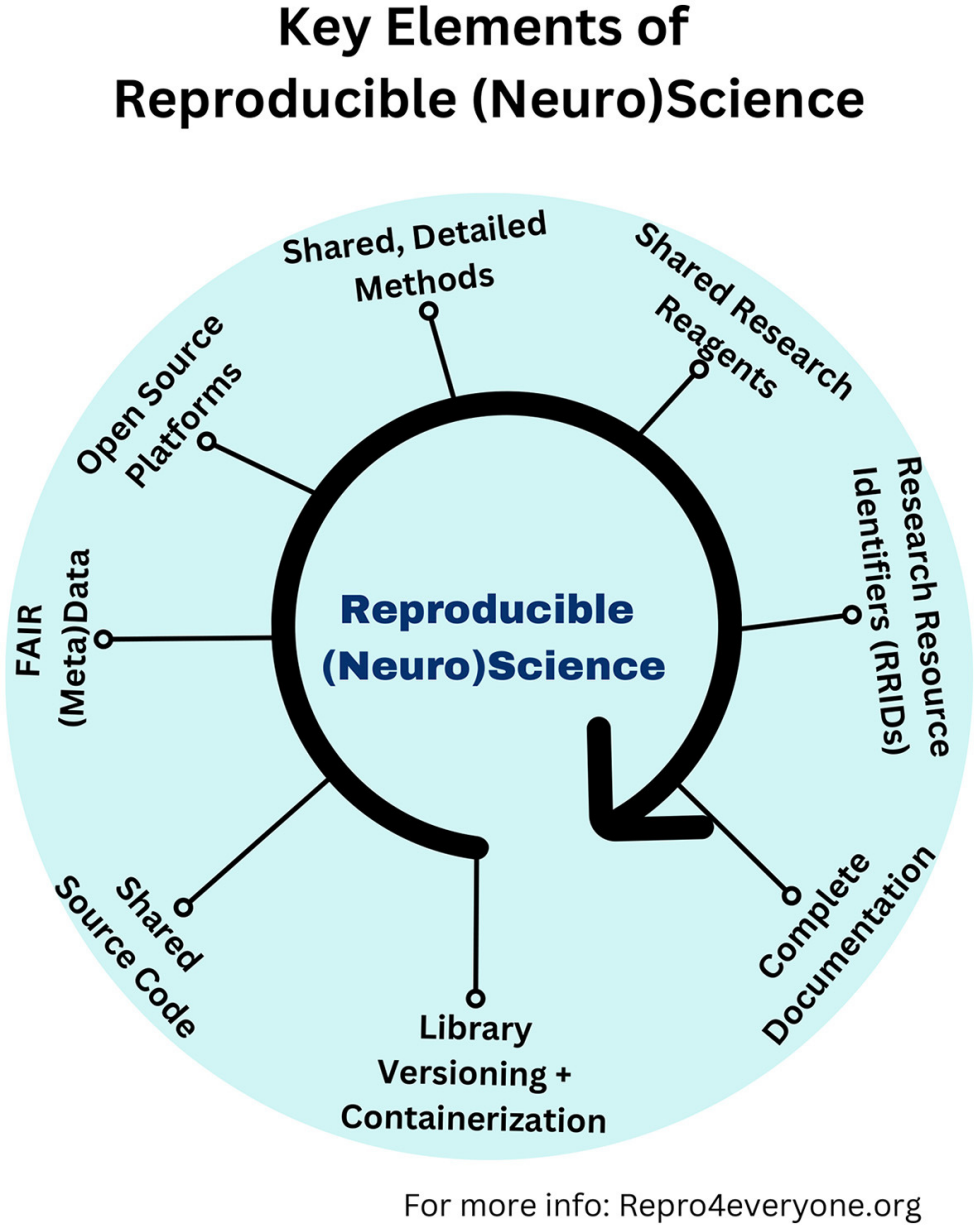
Key elements of reproducible (neuro)science. Graphical illustration showing which are the critical ingredients that make a paper or research project reproducible and replicable (Auer et al., 2021). Library versioning refers to the practice of assigning unique identifiers (usually numbers or names) to different releases or versions of a software library. Containerization is a technology that allows developers to package an application and all its dependencies (such as libraries and configurations) together into a single unit called a “container.” More information can be found at: www.repro4everyone.org.

A study by Wirth et al. measured vascular health in older adults. The study specifically aimed to replicate the link between resting state functional connectivity (RSFC) with functional brain networks (Köbe et al., 2021). The study examined 95 non-demented older adults from the IMAP+ cohort in France. The older adults had measurements taken at baseline, 18-, and 36-month time points. The researchers were able to replicate that RSFC increased over time. In addition, the scientists found that changes in RFSC also correlated with several measures of vascular health, such as diastolic blood pressure, β-Amyloid load, and glycated hemoglobin levels. The findings from this study show that good vascular health may help preserve brain health and cognitive resilience in older adults.

Moving toward the computational neuroscience realm, five of the papers included in this Research Topic used the open-source software NEST, a widespread spiking neural network (SNN) simulator, to reproduce prominent articles, with the additional benefit of implementing neural models using an up-to-date, reusable and maintainable simulation and analysis pipelines. All authors made the developed code publicly available, thus providing a more accessible version of the model to the computational neuroscience community.

Schulte to Brinke et al. successfully implemented the cortical column model originally proposed by Haeusler and Maass (2007). The results confirm the findings of the original study, most notably that the data-based circuit has superior computational performance to other control circuits without laminar structure. Going beyond the scope of the seminal work of Haeusler and Maass (2007), the study investigated the robustness with respect to the specifics of the neuron model used. To do so, Schulte to Brinke et al. reduced the complexity of the neuron model by eliminating intrinsic noise and simplifying it to an integrate-and-fire neuron.

The structure of the cortical columnar circuit was investigated by Zajzon et al. too, with a focus on cross-columnar communication. They used a SNN to conduct an extensive sensitivity analysis of the network originally implemented by Cone and Shouval (2021). They addressed the limits in biological plausibility found in the original model and proposed three alternative solutions.

Tiddia et al. replicated the simulations of working memory as proposed by Mongillo et al. (2008). While in the original study, the authors used a simple mean-field model to describe the firing rate behavior of an excitatory population modulated by short-term plasticity, Tiddia et al. created a SNN, which showed typical working memory behavior driven by short-term synaptic plasticity in a robust and energetically efficient manner.

Modeling and simulating a biologically relevant temporal component in neural networks to study spatiotemporal sequences observed in motor tasks has proven challenging, but a recent model developed by Maes et al. (2020) succeeded at creating a “neural clock” that can learn complex, higher-order sequences and behaviors. In a brief research report, Oberländer et al. re-implemented the SNN model and found they could replicate the original study's key findings.

Finally, Trapani et al. embedded the SNN cortical model proposed by Wang (2002) in a virtual robotic agent to perform a simulated behavioral task. They performed multiple simulations to assess the equivalence of the re-implemented SNN with the original study and validate its ability to perform an *in silico* behavioral task, discriminating between two stimuli, when embedded in a neurorobotics environment.

A study by Appukuttan and Davison explored reproducing a biologically-constrained point-neuron model of CA1 pyramidal neurons originally developed for Brian2 and NEURON simulators. The replication was purely based on the information contained within the published research article. The researchers found that they were able to replicate the core features of the model, but there were discrepancies that the authors could not account for, which might be a result of missing details in the original paper. The authors adopted the SciUnit framework (Omar et al., 2014), which offers a generalized approach that can be easily employed in other replication and reproduction studies.

In conclusion, the seven articles published in this Research Topic emphasize the strength and importance of replicating research studies to confirm and advance our knowledge in neuroscience. Independent review of the study design, source code, and/or research data is essential for confirming the robustness and generalizability of the original findings and for building on them to further advance our knowledge. In addition, the re-introduction and adoption of computational models onto open-source platforms, such as NEST, help make the models more accessible to the broader research community. However, care should be taken when editing and reviewing replication studies as we have found that not every researcher understands the need and importance of publishing replication studies. In addition, while it may seem logical to invite the authors of the original study to review the study, their underlying bias may result in some issues, both in the case of confirmatory or contrasting results. We hope this Research Topic and editorial demonstrate the importance and value of reproducibility and replicability in (neuro)science.

## Author contributions

NJ: Writing—original draft, Writing—review and editing. NH: Writing—original draft, Writing—review and editing. RS: Writing—original draft, Writing—review and editing. AA: Conceptualization, Supervision, Writing—original draft, Writing—review and editing.
